# Supplementary material for: Deposition and water repelling of temperature-responsive nanopesticides on leaves
Source: Nat Commun. 2023 Oct 12;14:6401. doi: 10.1038/s41467-023-41878-3 (PMC10570302; doi:10.1038/s41467-023-41878-3)
Supplement: Supplementary file 1 — Supplementary Information [file 41467_2023_41878_MOESM1_ESM.pdf]

Supplementary Information for Deposition and Water Repelling of Temperature-Responsive Nanopesticides on Leaves

Jie Tang[a], Xiaojing Tong[a], Yongjun Chen[b], Yue Wu[a], Zhiyuan Zheng[a], A. Basak, Kayitmazer[c], Ayyaz Ahmad[d], Naveed Ramzan[e], Jintao Yang[f], Qingchun Huang[b], Yisheng Xu*, [a]

[a] State Key Laboratory of Chemical Engineering, East China University of Science and Technology, Shanghai 200237, P. R. China.

Email: yshxu@ecust.edu.cn

[b] Shanghai Key Lab of Chemical Biology, School of Pharmacy, East China University of Science and Technology, Shanghai 200237, P. R. China

[c] Department of Chemistry, Bogazici University, Istanbul, Turkey

[d] Department of Chemical Engineering, Muhammad Nawaz Sharif University of Engineering and Technology, Multan, Pakistan

[e] Faculty of Chemical, Metallurgical, and Polymer Engineering, University of Engineering & Technology, Lahore, Pakistan

[f] College of Materials Science & Engineering, Zhejiang University of Technology, Hangzhou 310014, P. R. China

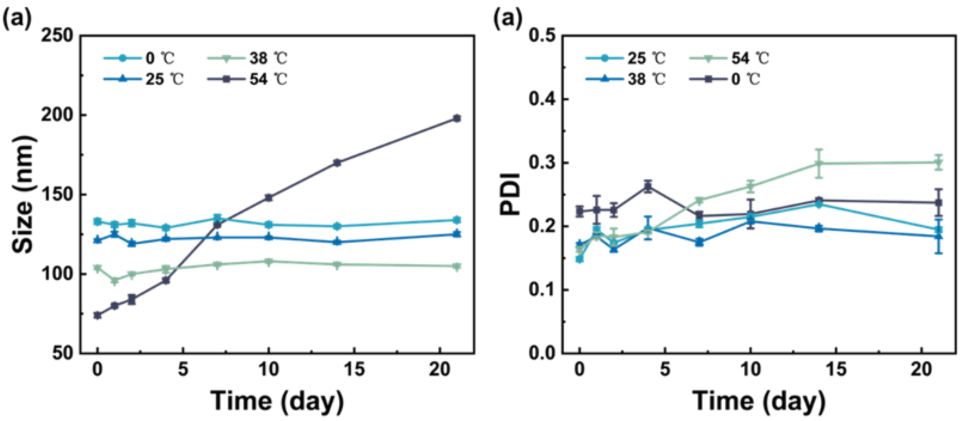

Supplementary Figure 1. the stability of (a) size and (b) PDI of TEB NPs at 0 °C, 25 °C, 38 °C, and 54 °C (n = 3 independent experiments, Data are presented as mean values ± SD)

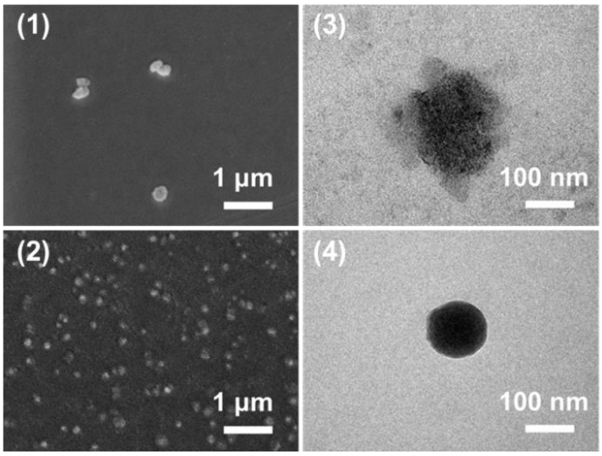

Supplementary Figure 2. SEM images of TEB NPs prepared at (1) 2 mL/min and (2) 30 mL/min; TEM images of TEB NPs prepared at (3) 2 mL/min and (4) 30 mL/min (each experiment was repeated three times independently with similar results)

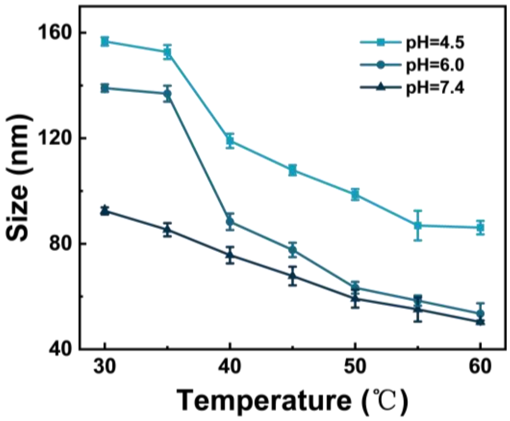

Supplementary Figure 3. The size of TEB NPs at different temperature and pH with 0.05 mg/mL PDMAEMA-*b*-PCL and 0.1 mg/mL TEB (n = 3 independent experiments, Data are presented as mean values ± SD)

Supplementary Table 1. Fitting results for the data of photodegradation of varying samples

Fitting formula	Sample	k (h^{-1})	R^2	$t_{1/2}$(h)
$y = exp^{-kt}$	TEB NPs	0.0498±0.0037	0.9272	13.9158
	SC	0.0857±0.0065	0.9392	8.0900
	WP	0.1065±0.0129	0.8459	6.5109
	Free TEB	0.13820±0.0187	0.8419	5.0159

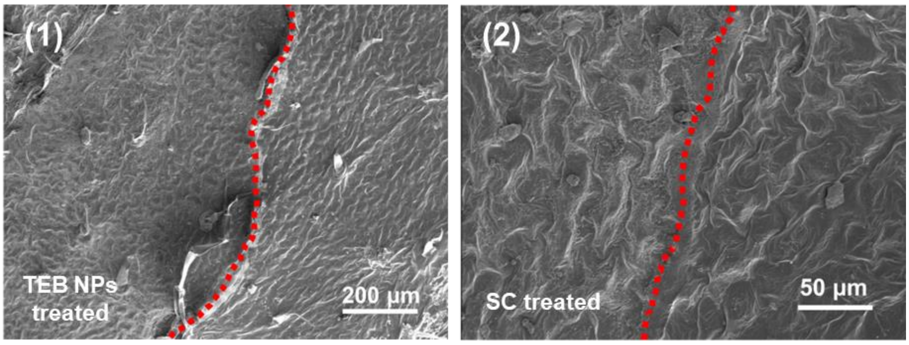

Supplementary Figure 4. SEM images of tomato leaves coated (1) TEB NPs, and (2) commercial TEB formulation (SC). For clarity, the image was taken near the borderline between TEB formulation coating area and bare tomato leaf surface (as indicated by a curve) (each experiment was repeated three times independently with similar results)

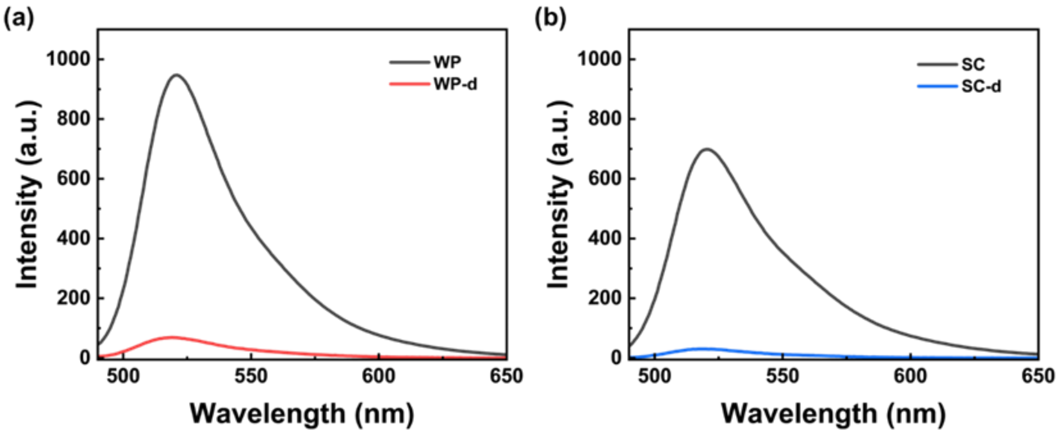

Supplementary Figure 5. Fluorescence spectra of (a) commercial WP and dialysate of commercial WP, and (b) commercial SC and dialysate of commercial SC

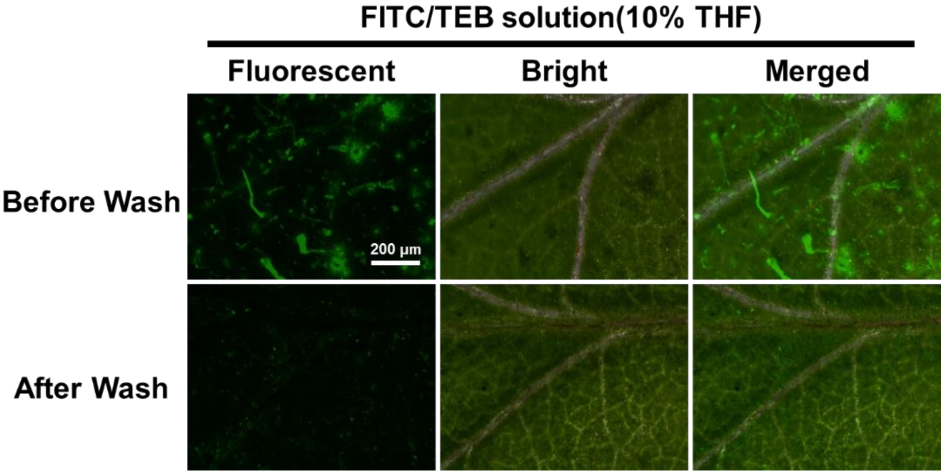

Supplementary Figure 6. Fluorescence images of TEB solution (10% THF) on the tomato leaves to simulate rain washing resistance (Scale bar = 200 μm, each experiment was repeated three times independently with similar results)

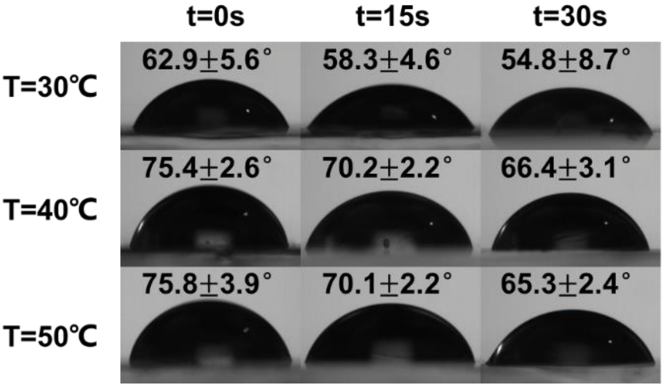

Supplementary Figure 7. The contact angle of TEB NPs at different temperature on the surface of glass within 0, 15, and 30 seconds (n = 3 independent experiments)

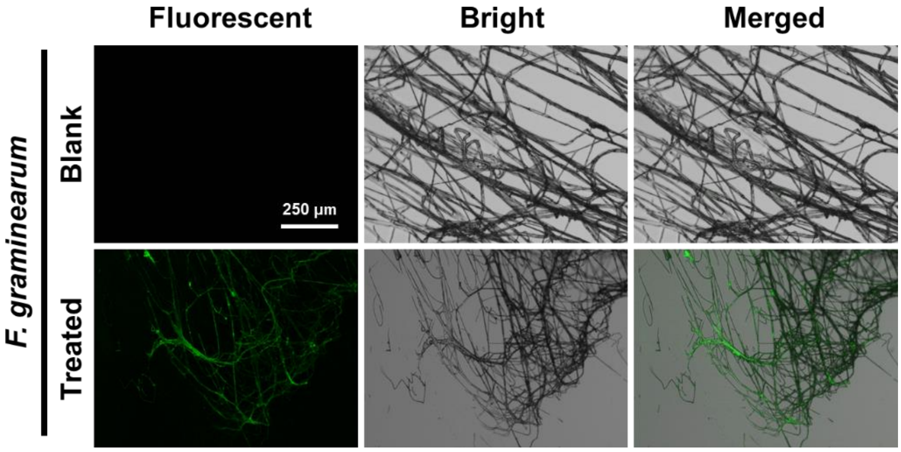

Supplementary Figure 8. CLSM images of mycelia of wheat fusarium head blight (*F. graminearum*) (each experiment was repeated three times independently with similar results)

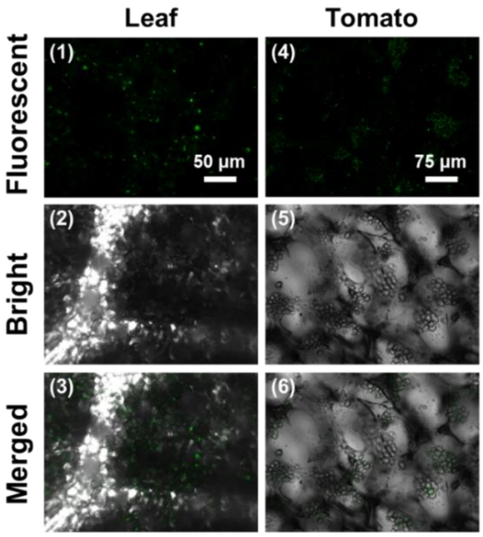

Supplementary Figure 9. (a) CLSM images of (1-3) the untreated section of the leaf adjacent to the treated position, and (4-6) the untreated section of the tomato flesh portion adjacent to the treated position (each experiment was repeated three times independently with similar results)

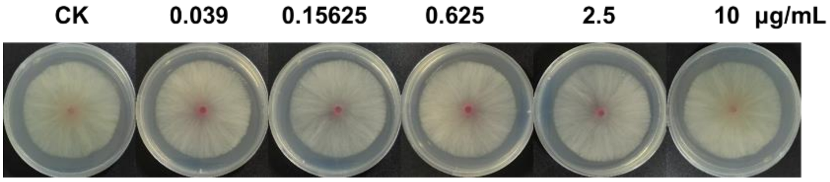

Supplementary Figure 10. Images of the fungicidal activity of PDMAEMA-*b*-PCL against *F. graminearum* (wheat fusarium head blight) after the 3d-treatment. The concentration of polymer varied from 0.039, 0.156, 0.625, 2.5 to 10 μg/mL

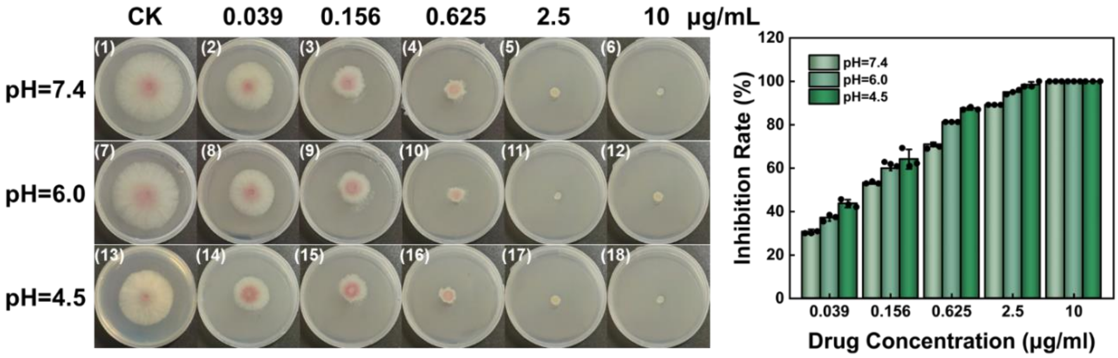

Supplementary Figure 11. Images of the fungicidal activity and inhibition rate of TEB NPs against *F. graminearum* (wheat fusarium head blight) at different pHs after the 3d-treatment. The concentration of polymer varied from 0.039, 0.156, 0.625, 2.5 to 10 μg/mL (n = 3 independent experiments, Data are presented as mean values ± SD)

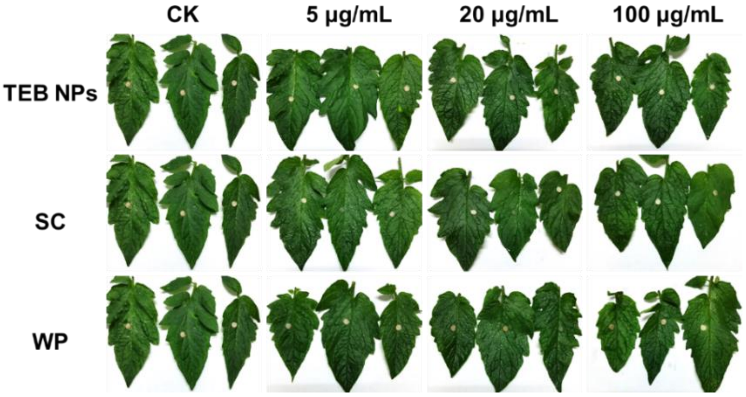

Supplementary Figure 12. Protective activities of different TEB formulations against *B. cinerea* on tomato leaves after the 1d-treatment (n = 3 independent experiments)

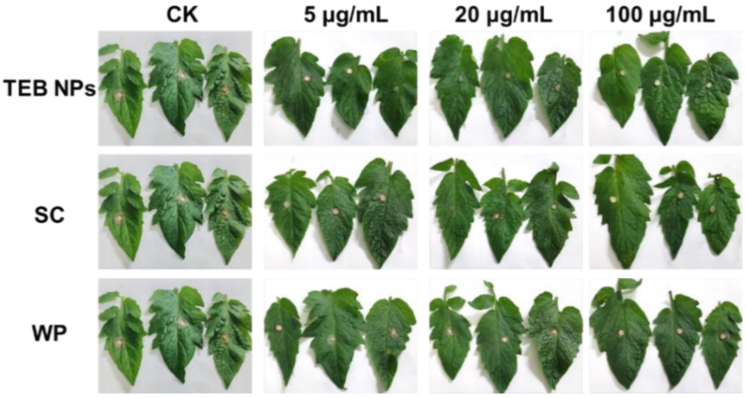

Supplementary Figure 13. Protective activities of different TEB formulations against *B. cinerea* on tomato leaves after the 2d-treatment (n = 3 independent experiments)

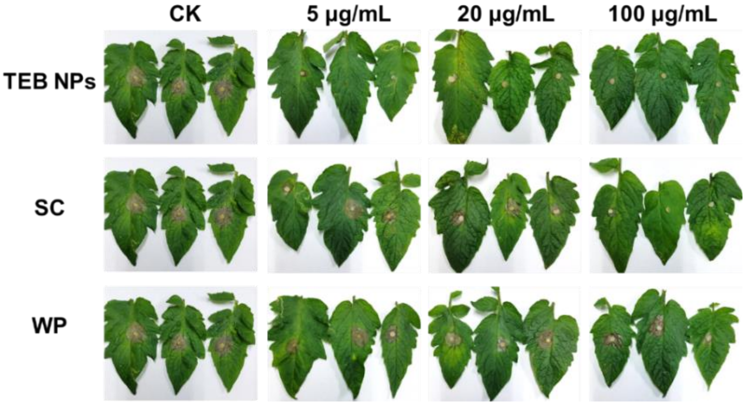

Supplementary Figure 14. Protective activities of different TEB formulations against *B. cinerea* on tomato leaves after the 3d-treatment (n = 3 independent experiments)

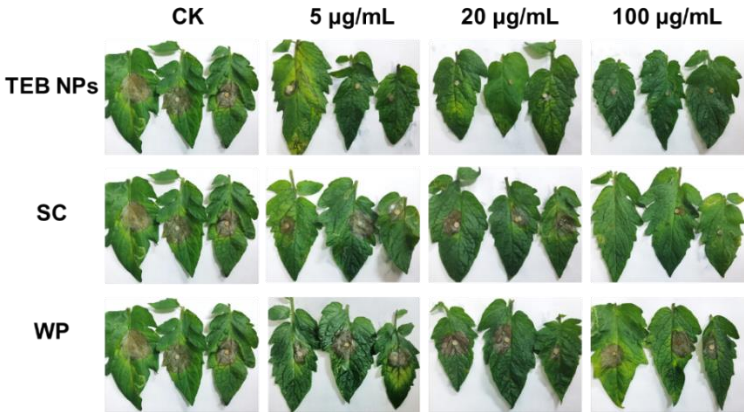

Supplementary Figure 15. Protective activities of different TEB formulations against *B. cinerea* on tomato leaves after the 4d-treatment (n = 3 independent experiments)

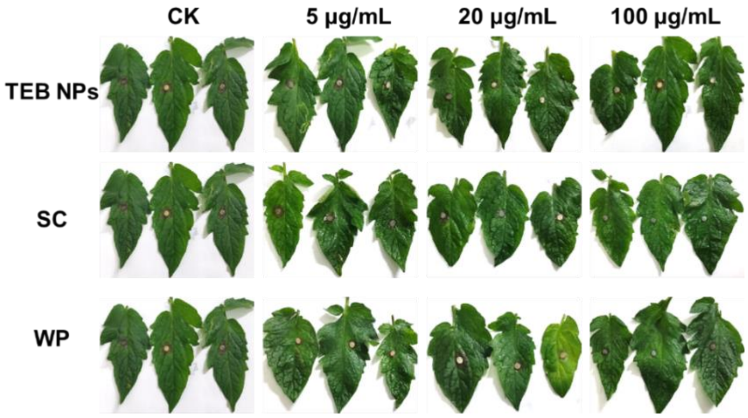

Supplementary Figure 16. Curative activities of different TEB formulations against *B. cinerea* on tomato leaves after the 1d-treatment (n = 3 independent experiments)

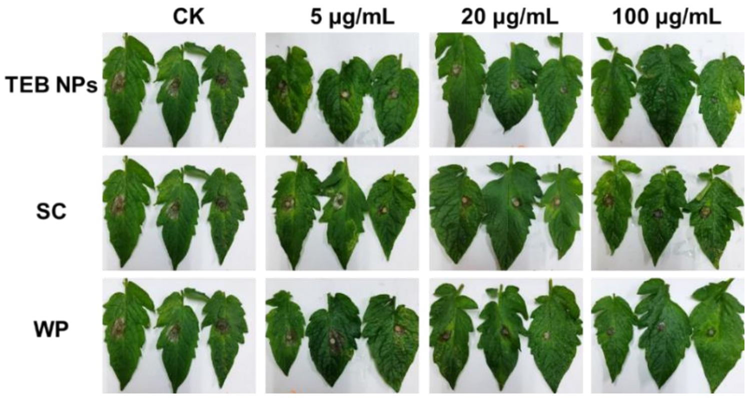

Supplementary Figure 17. Curative activities of different TEB formulations against *B. cinerea* on tomato leaves after the 2d-treatment (n = 3 independent experiments)

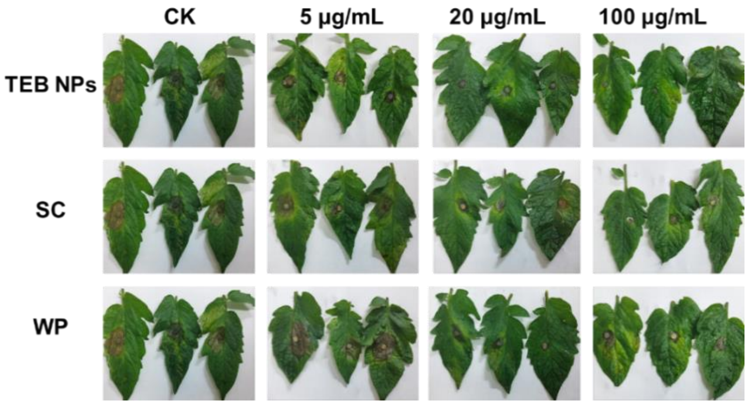

Supplementary Figure 18. Curative activities of different TEB formulations against *B. cinerea* on tomato leaves after the 3d-treatment (n = 3 independent experiments)

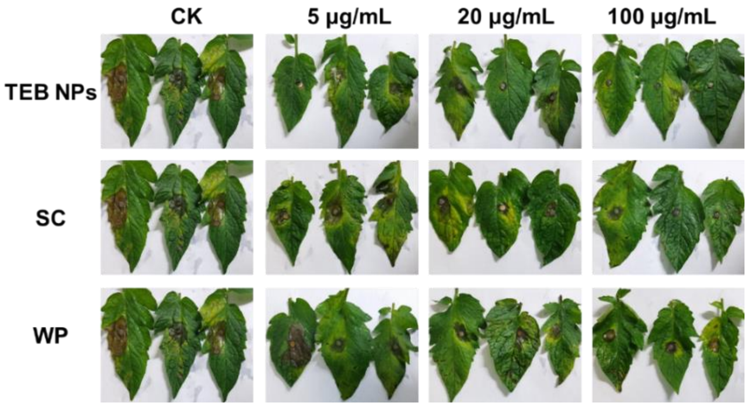

Supplementary Figure 19. Curative activities of different TEB formulations against *B. cinerea* on tomato leaves after the 4d-treatment (n = 3 independent experiments)

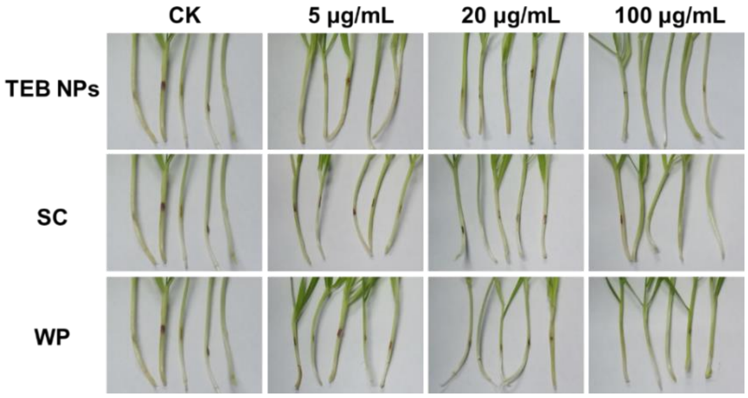

Supplementary Figure 20. Protective activities of different TEB formulations against *F. graminearum* on wheat coleoptile after the 1d-treatment (n = 5 independent experiments)

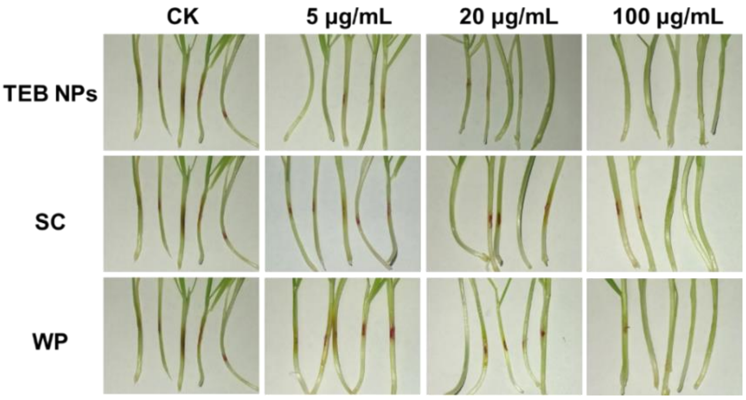

Supplementary Figure 21. Protective activities of different TEB formulations against *F. graminearum* on wheat coleoptile after the 2d-treatment (n = 5 independent experiments)

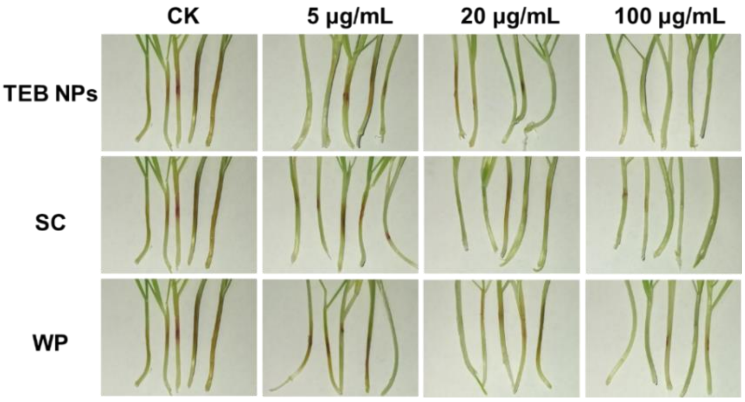

Supplementary Figure 22. Protective activities of different TEB formulations against *F. graminearum* on wheat coleoptile after the 3d-treatment (n = 5 independent experiments)

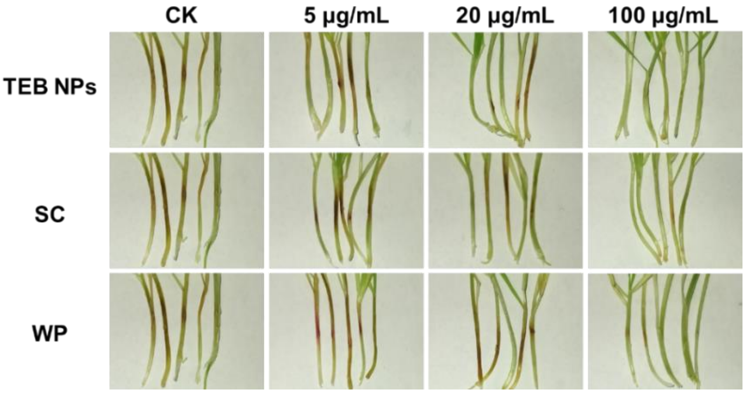

Supplementary Figure 23. Protective activities of different TEB formulations against *F. graminearum* on wheat coleoptile after the 4d-treatment (n = 5 independent experiments)

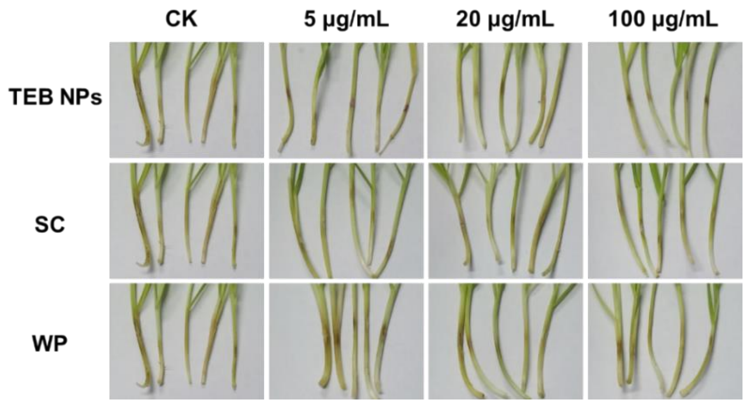

Supplementary Figure 24. Curative activities of different TEB formulations against *F. graminearum* on wheat coleoptile after the 1d-treatment (n = 5 independent experiments)

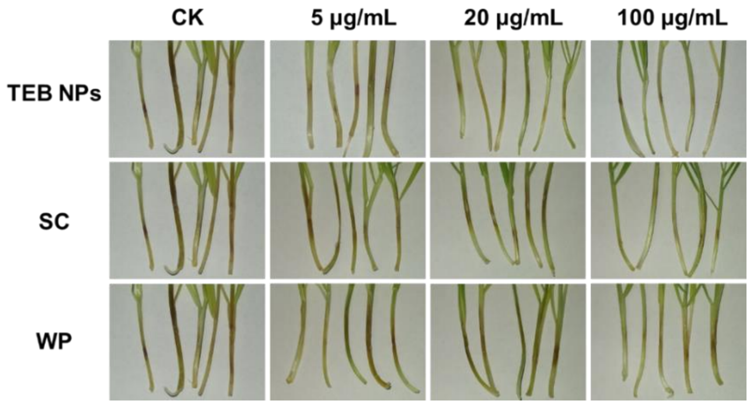

Supplementary Figure 25. Curative activities of different TEB formulations against *F. graminearum* on wheat coleoptile after the 2d-treatment (n = 5 independent experiments)

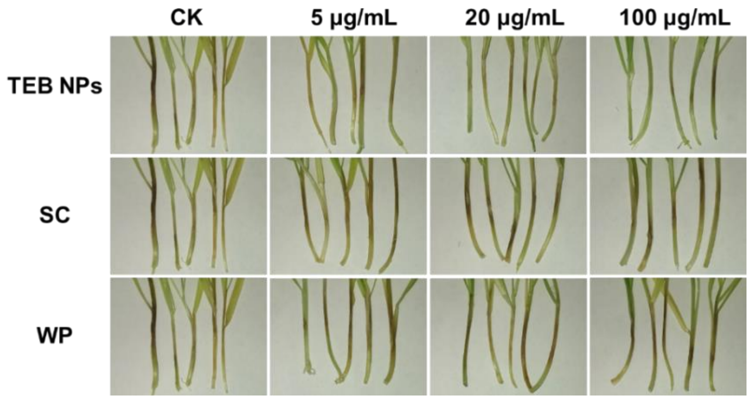

Supplementary Figure 26. Curative activities of different TEB formulations against *F. graminearum* on wheat coleoptile after the 3d-treatment (n = 5 independent experiments)

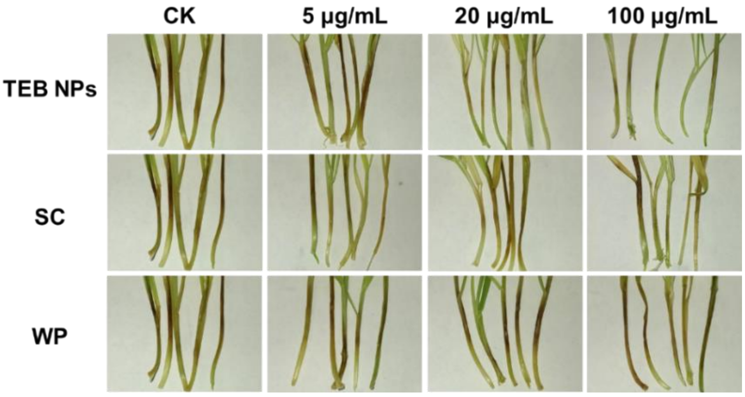

Supplementary Figure 27. Curative activities of different TEB formulations against *F. graminearum* on wheat coleoptile after the 4d-treatment (n = 5 independent experiments)

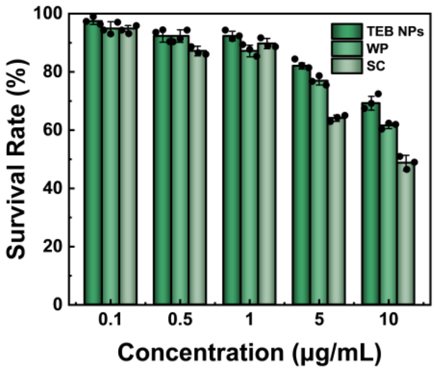

Supplementary Figure 28. The survival rate of zebrafish larvae (5 dpf) exposed to different TEB formulations with concentrations of 0.1, 0.5, 1, 5, 10 μg/mL at 24 hr (n = 15 larvae examined over 3 independent experiments, Data are presented as mean values ± SD)

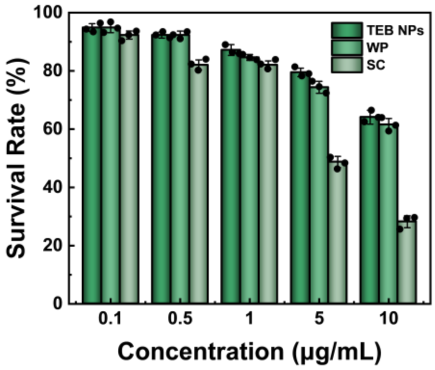

Supplementary Figure 29. The survival rate of zebrafish larvae (5 dpf) exposed to different TEB formulations with concentrations of 0.1, 0.5, 1, 5, 10 μg/mL at 48 hr (n = 15 larvae examined over 3 independent experiments, Data are presented as mean values ± SD)

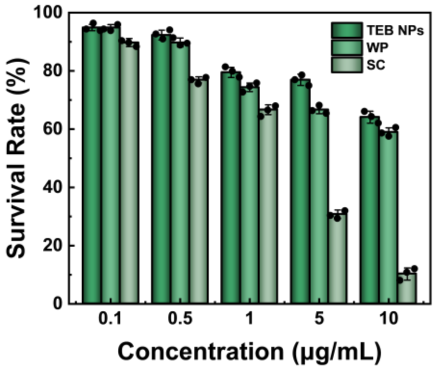

Supplementary Figure 30. The survival rate of zebrafish larvae (5 dpf) exposed to different TEB formulations with concentrations of 0.1, 0.5, 1, 5, 10 μg/mL at 72 hr (n = 15 larvae examined over 3 independent experiments, Data are presented as mean values ± SD)

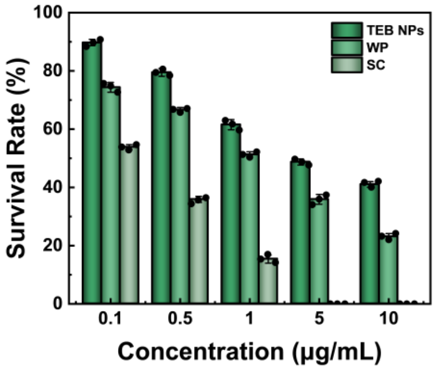

Supplementary Figure 31. The survival rate of zebrafish larvae (5 dpf) exposed to different TEB formulations with concentrations of 0.1, 0.5, 1, 5, 10 μg/mL at 120 hr (n = 15 larvae examined over 3 independent experiments, Data are presented as mean values ± SD)

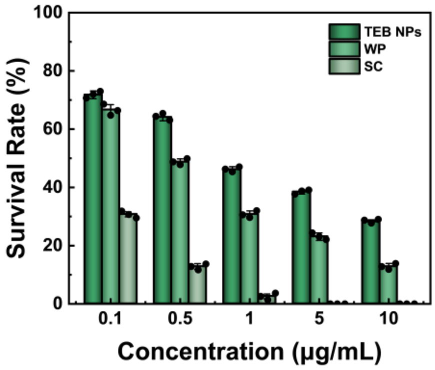

Supplementary Figure 32. The survival rate of zebrafish larvae (5 dpf) exposed to different TEB formulations with concentrations of 0.1, 0.5, 1, 5, 10 μg/mL at 144 hr (n = 15 larvae examined over 3 independent experiments, Data

are presented as mean values ± SD)

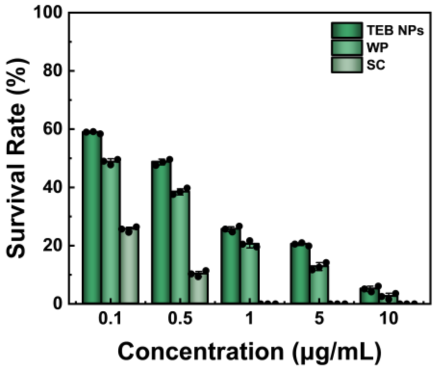

Supplementary Figure 33. The survival rate of zebrafish larvae (5 dpf) exposed to different TEB formulations with concentrations of 0.1, 0.5, 1, 5, 10 μg/mL at 168 hr (n = 15 larvae examined over 3 independent experiments, Data are presented as mean values ± SD

Supplementary Table 2. The LC_{50} values of different TEB formulations to zebrafish larvae

Samples	Time (hr)	LC_{50} (μg/mL)	99% confidence interval	R^2	Equation
TEB NPs	24	68.697	20.849-226.353	0.9628	y = 0.6791x + 3.7018
	48	55.931	17.475-179.021	0.9370	y = 0.6097x + 3.8758
	72	47.599	14.759-153.517	0.9014	y = 0.6211x + 3.9546
	96	12.813	6.342-25.888	0.9423	y = 0.6440x + 4.2723
	120	4.338	2.807-6.701	0.9659	y = 0.7589x + 4.5146
	144	1.174	0.745-1.850	0.9456	y = 0.5751x + 4.9601
	168	0.248	0.158-0.391	0.8891	y = 0.8609x + 5.5146
WP	24	38.692	14.628-102.344	0.9442	y = 0.6553x + 3.9032
	48	33.563	13.235-85.112	0.9572	y = 0.6717x + 3.9412
	72	19.070	8.690-41.849	0.9373	y = 0.7149x + 4.1008
	96	4.583	2.848-7.375	0.9622	y = 0.6848x + 4.5414
	120	1.234	0.843-1.806	0.9601	y = 0.6966x + 4.9406
	144	0.359	0.234-0.553	0.9652	y = 0.7511x + 5.3352
	168	0.120	0.067-0.214	0.9136	y = 0.8723x + 5.7635
SC	24	11.928	7.046-20.193	0.9208	y = 0.8248x + 4.0616
	48	4.058	2.940-5.601	0.9372	y = 0.9863x + 4.3911
	72	1.667	1.311-2.119	0.9553	y = 1.2404x + 4.7684
	96	0.449	0.337-0.598	0.8919	y = 1.3520x + 5.5112
	120	0.155	0.108-0.221	0.9529	y = 1.3612x + 6.0791
	144	0.032	0.015-0.070	0.9129	y = 0.9628x + 6.5712
	168	0.020	0.007-0.053	0.7891	y = 0.8668x + 6.7111

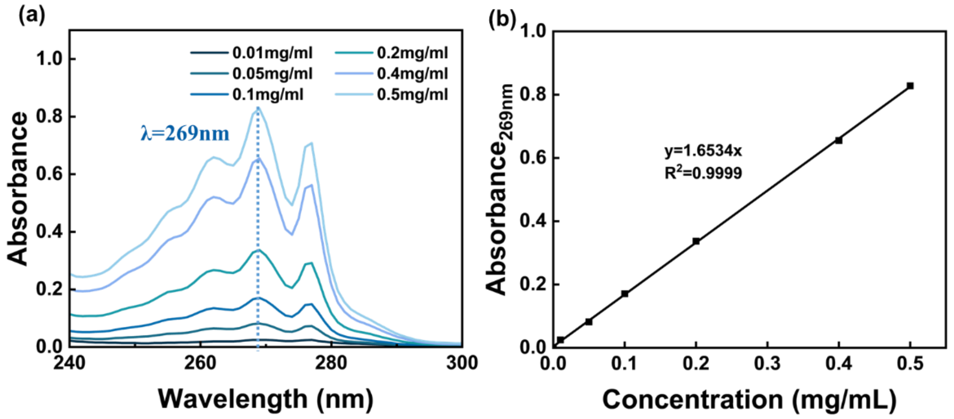

Supplementary Figure 34. (a) Absorbance spectrum and (b) standard calibration curve of tebuconazole with various concentrations in the solution of THF/water = 90: 10 v/v

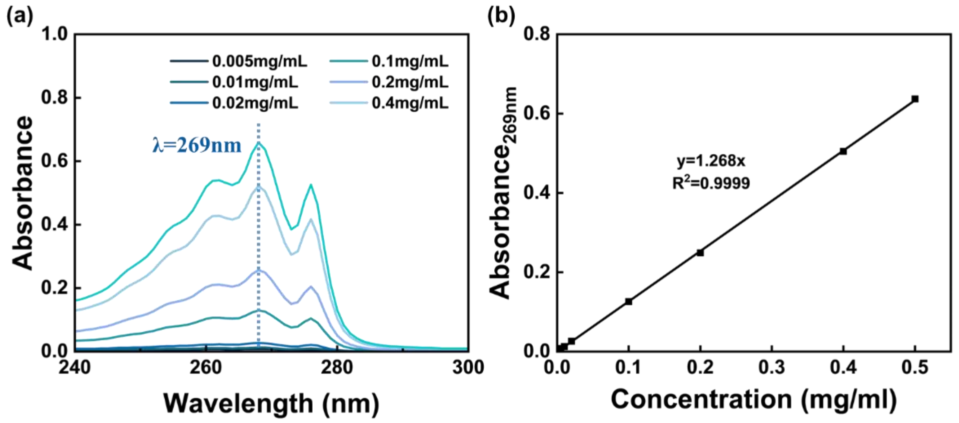

Supplementary Figure 35. (a) Absorbance spectrum and (b) standard calibration curve of tebuconazole with various concentrations in the solution of ethanol/water = 20:80 v/v
