# Supplementary material for: Deposition and water repelling of temperature-responsive nanopesticides on leaves
Source: Nat Commun. 2023 Oct 12;14:6401. doi: 10.1038/s41467-023-41878-3 (PMC10570302; doi:10.1038/s41467-023-41878-3)
Supplement: Supplementary file 3 — Reporting Summary [file 41467_2023_41878_MOESM3_ESM.pdf]

nature portfolio

Corresponding author(s): Yisheng Xu

Last updated by author(s): Sep 3, 2023

# Reporting Summary

Nature Portfolio wishes to improve the reproducibility of the work that we publish. This form provides structure for consistency and transparency in reporting. For further information on Nature Portfolio policies, see our Editorial Policies and the Editorial Policy Checklist.

## Statistics

For all statistical analyses, confirm that the following items are present in the figure legend, table legend, main text, or Methods section.

| n/a | Confirmed | |
|---|---|---|
| ☐ | ☒ | The exact sample size ($n$) for each experimental group/condition, given as a discrete number and unit of measurement |
| ☐ | ☒ | A statement on whether measurements were taken from distinct samples or whether the same sample was measured repeatedly |
| ☐ | ☒ | The statistical test(s) used AND whether they are one- or two-sided<br>*Only common tests should be described solely by name; describe more complex techniques in the Methods section.* |
| ☒ | ☐ | A description of all covariates tested |
| ☒ | ☐ | A description of any assumptions or corrections, such as tests of normality and adjustment for multiple comparisons |
| ☐ | ☒ | A full description of the statistical parameters including central tendency (e.g. means) or other basic estimates (e.g. regression coefficient) AND variation (e.g. standard deviation) or associated estimates of uncertainty (e.g. confidence intervals) |
| ☒ | ☐ | For null hypothesis testing, the test statistic (e.g. $F$, $t$, $r$) with confidence intervals, effect sizes, degrees of freedom and $P$ value noted<br>*Give P values as exact values whenever suitable.* |
| ☒ | ☐ | For Bayesian analysis, information on the choice of priors and Markov chain Monte Carlo settings |
| ☒ | ☐ | For hierarchical and complex designs, identification of the appropriate level for tests and full reporting of outcomes |
| ☒ | ☐ | Estimates of effect sizes (e.g. Cohen's $d$, Pearson's $r$), indicating how they were calculated |

*Our web collection on statistics for biologists contains articles on many of the points above.*

## Software and code

Policy information about availability of computer code

| | |
|---|---|
| Data collection | image J software (image J 1.53u, U.S.), Origin software (Origin 2023 Corporation, U.S.) |
| Data analysis | image J software (image J 1.53u, U.S.), Origin software (Origin 2023 Corporation, U.S.) |

For manuscripts utilizing custom algorithms or software that are central to the research but not yet described in published literature, software must be made available to editors and reviewers. We strongly encourage code deposition in a community repository (e.g. GitHub). See the Nature Portfolio guidelines for submitting code & software for further information.

## Data

Policy information about availability of data

All manuscripts must include a data availability statement. This statement should provide the following information, where applicable:

- Accession codes, unique identifiers, or web links for publicly available datasets
- A description of any restrictions on data availability
- For clinical datasets or third party data, please ensure that the statement adheres to our policy

The source data generated in this study are provided in the Source Data file.

# Research involving human participants, their data, or biological material

Policy information about studies with human participants or human data. See also policy information about sex, gender (identity/presentation), and sexual orientation and race, ethnicity and racism.

| | |
|---|---|
| Reporting on sex and gender | n/a |
| Reporting on race, ethnicity, or other socially relevant groupings | n/a |
| Population characteristics | n/a |
| Recruitment | n/a |
| Ethics oversight | n/a |

Note that full information on the approval of the study protocol must also be provided in the manuscript.

# Field-specific reporting

Please select the one below that is the best fit for your research. If you are not sure, read the appropriate sections before making your selection.

☐ Life sciences ☐ Behavioural & social sciences ☒ Ecological, evolutionary & environmental sciences

For a reference copy of the document with all sections, see nature.com/documents/nr-reporting-summary-flat.pdf

# Ecological, evolutionary & environmental sciences study design

All studies must disclose on these points even when the disclosure is negative.

| | |
|---|---|
| Study description | In this study, we have successfully prepared leaf-adhesive, water-stable, small particle size, and well-dispersed tebuconazole (an ergosterol synthesis inhibitor, TEB) NPs using the FNP method with the block copolymer poly-(2-(dimethylamino)ethylmethylacrylate)-b-poly(ε-caprolactone) (PDMAEMA-b-PCL) as the carrier, which has charged and temperature-responsive groups26 allowing the efficient disposition of NPs at low temperatures and with good water repelling properties as the temperature is increased, In this study, we have successfully prepared leaf-adhesive, water-stable, small particle size, and well-dispersed tebuconazole (an ergosterol synthesis inhibitor, TEB) NPs using the FNP method with the block copolymer poly-(2-(dimethylamino)ethylmethylacrylate)-b-poly(ε-caprolactone) (PDMAEMA-b-PCL) as the carrier, which has charged and temperature-responsive groups allowing the efficient disposition of NPs at low temperatures and with good water repelling properties as the temperature is increased. |
| Research sample | Selection of live tomato leaves and wheat coleoptile was determined on the basis of size and growth status, Live tomato leaves of similar size and growth status were obtained from a farm in Shanghai, and wheat coleoptile of uniform growth was planted evenly in our laboratory. Commercial TEB formulations were set according to the popularity and sales volume of the formulation.<br>To evaluate the acute toxicity of TEB NPs to aquatic organisms, zebrafish were selected as model animal. WT line wild-type zebrafish larvae were obtained from the Laboratory of Aquatic Animal Diseases of East China University of Science and Technology (Shanghai, China), and maintained with E3 medium according to the standard protocol. For safety assessment in zebrafish, 5 d postfertilization (dpf), zebrafish larvae (15 larvae per treatment) were randomly picked and immersed in a six-well plate with different TEB formulations at the concentration of 0.1, 0.5, 1, 5, 10 μg/mL. During the exposure, all the fish were unfed. Zebrafish deaths were recorded at the indicated time points. Each exposure was repeated thrice. |
| Sampling strategy | To evaluate the acute toxicity of TEB NPs to aquatic organisms, zebrafish were selected as model animal. WT line wild-type zebrafish larvae were obtained from the Laboratory of Aquatic Animal Diseases of East China University of Science and Technology (Shanghai, China), and maintained with E3 medium according to the standard protocol. For safety assessment in zebrafish, 5 d postfertilization (dpf), zebrafish larvae (15 larvae per treatment) were randomly picked and immersed in a six-well plate with different TEB formulations at the concentration of 0.1, 0.5, 1, 5, 10 μg/mL. During the exposure, all the fish were unfed. Zebrafish deaths were recorded at the indicated time points. Each exposure was repeated thrice. |
| Data collection | the size of nano-pesticides was recorded by Jie Tang and Xiaojing Tong using NICOMP 380 ZLS. The average of three measurements was reported. |
| Timing and spatial scale | The zebrafish experiment began on June 1, 2023, and ceased on June 8, 2023, with sampling at 24-hour intervals. The frequency and periodicity of sampling was set according to the experience and relevant literature. |
| Data exclusions | no data were excluded from the analysis. |
| Reproducibility | All the treatments were performed 3-6 times. |
| Randomization | the allocation of tomato leaves, wheat coleoptile and zebrafish larvae was random. |

| | |
|---|---|
| Blinding | Because our experiments involved only zebrafish larvae and plants, there was no subjective human influence on the experimental organisms, and there is no blinding in our experiments. |

Did the study involve field work? ☐ Yes ☒ No

# Reporting for specific materials, systems and methods

We require information from authors about some types of materials, experimental systems and methods used in many studies. Here, indicate whether each material, system or method listed is relevant to your study. If you are not sure if a list item applies to your research, read the appropriate section before selecting a response.

## Materials & experimental systems

| n/a | Involved in the study | |
|---|---|---|
| ☒ | ☐ | Antibodies |
| ☒ | ☐ | Eukaryotic cell lines |
| ☒ | ☐ | Palaeontology and archaeology |
| ☐ | ☒ | Animals and other organisms |
| ☒ | ☐ | Clinical data |
| ☒ | ☐ | Dual use research of concern |
| ☐ | ☒ | Plants |

## Methods

| n/a | Involved in the study | |
|---|---|---|
| ☒ | ☐ | ChIP-seq |
| ☒ | ☐ | Flow cytometry |
| ☒ | ☐ | MRI-based neuroimaging |

# Animals and other research organisms

Policy information about studies involving animals; ARRIVE guidelines recommended for reporting animal research, and Sex and Gender in Research

| | |
|---|---|
| Laboratory animals | WT line wild-type zebrafish larvae were obtained from the State Key Laboratory of Bioreactor Engineering of East China University of Science and Technology (Shanghai, China), and maintained with E3 medium according to the standard protocol. |
| Wild animals | the study did not involve wild animals. |
| Reporting on sex | n/a |
| Field-collected samples | The susceptible Botrytis cinerea (a pathogen that causes gray mold) was collected from tomato fields, and Fusarium graminearum (a pathogen that causes fusarium head blight) was collected from wheat fields in Shanghai suburbs. They were identified, isolated, and conserved by our laboratory. The isolates were grown on potato dextrose agar (PDA) at 25 ± 2 ℃ in routine assays to assess the antifungal action of TEB in vitro and in vivo. |
| Ethics oversight | All zebrafish experiments were conformed to the Zebrafish Information Network guidelines for the care and use of laboratory animals and ethically approved by the Laboratory Animal Ethical Committee of East China University of Science and Technology (Protocol number 2006272). |

Note that full information on the approval of the study protocol must also be provided in the manuscript.

# Dual use research of concern

Policy information about dual use research of concern

## Hazards

Could the accidental, deliberate or reckless misuse of agents or technologies generated in the work, or the application of information presented in the manuscript, pose a threat to:

| No | Yes | |
|---|---|---|
| ☒ | ☐ | Public health |
| ☒ | ☐ | National security |
| ☒ | ☐ | Crops and/or livestock |
| ☒ | ☐ | Ecosystems |
| ☒ | ☐ | Any other significant area |

## Experiments of concern

Does the work involve any of these experiments of concern:

| No | Yes | |
|---|---|---|
| ☒ | ☐ | Demonstrate how to render a vaccine ineffective |
| ☒ | ☐ | Confer resistance to therapeutically useful antibiotics or antiviral agents |
| ☒ | ☐ | Enhance the virulence of a pathogen or render a nonpathogen virulent |
| ☒ | ☐ | Increase transmissibility of a pathogen |
| ☒ | ☐ | Alter the host range of a pathogen |
| ☒ | ☐ | Enable evasion of diagnostic/detection modalities |
| ☒ | ☐ | Enable the weaponization of a biological agent or toxin |
| ☒ | ☐ | Any other potentially harmful combination of experiments and agents |
